# Facile preparation of toluidine blue-loaded DNA nanogels for anticancer photodynamic therapy

**DOI:** 10.3389/fbioe.2023.1180448

**Published:** 2023-04-18

**Authors:** Hua Guo, Huimin Wang, Hong Deng, Yiyi Zhang, Xue Yang, Weiqi Zhang

**Affiliations:** ^1^ National Cancer Center/National Clinical Research Center for Cancer/Cancer Hospital, Chinese Academy of Medical Sciences and Peking Union Medical College, Beijing, China; ^2^ State Key Laboratory of Medical Molecular Biology and Department of Biomedical Engineering, Institute of Basic Medical Sciences, Chinese Academy of Medical Sciences and Peking Union Medical College, Beijing, China

**Keywords:** DNA nanogel, photodynamic therapy, toluidine blue, cancer therapy, self-assembly

## Abstract

Photodynamic therapy (PDT) provides an effective therapeutic option for different types of cancer in addition to surgery, radiation, and chemotherapy. The treatment outcome of PDT is largely determined by both the light and dark toxicity of photosensitizers (PSs), which can be technically improved with the assistance of a drug delivery system, especially the nanocarriers. Toluidine blue (TB) is a representative PS that demonstrates high PDT efficacy; however, its application is largely limited by the associated dark toxicity. Inspired by TB’s noncovalent binding with nucleic acids, in this study, we demonstrated that DNA nanogel (NG) could serve as an effective TB delivery vehicle to facilitate anticancer PDT. The DNA/TB NG was constructed by the simple self-assembly between TB and short DNA segments using cisplatin as a crosslinker. Compared with TB alone, DNA/TB NG displayed a controlled TB-releasing behavior, effective cellular uptake, and phototoxicity while reducing the dark toxicity in breast cancer cells MCF-7. This DNA/TB NG represented a promising strategy to improve TB-mediated PDT for cancer treatments.

## 1 Introduction

Photodynamic therapy (PDT) for cancers has gained increasing research interest due to its advantages, such as light-controlled toxicity, minimal invasiveness, feasibility of repeated treatments, and the potential to elicit anticancer immune responses (Agostinis et al., 2011; Li et al., 2020). With the presence of molecular oxygen and light, a photosensitizer (PS) generates reactive oxygen species (ROS) that can destroy the cell components, including DNA, proteins, and lipids, which collectively causes cell death (Pham et al., 2021). However, because of the intrinsic properties of PSs, PDT also suffers from several disadvantages, such as limited light penetration, poor solubility, and lack of cancer-targeting capability. Intensive efforts were made to improve the anticancer PDT performance including synthesizing near-infrared PS to obtain better light penetration, engineering the PS structure to improve the bioavailability and PDT efficiency, and encapsulating PSs in carriers to target the tumor cells (Wang et al., 2016; Pham et al., 2021; Marino-Ocampo et al., 2022). Generally, the ideal cancer PDT relies on an optimized light/dark toxicity ratio of PS, corresponding to a precise phototoxicity of tumor and a minimized side effect.

Toluidine blue (TB) is a cationic phenothiazinium dye that has been widely used as PS due to its near-infrared absorption (∼630 nm), good water solubility, and high ROS generation ability (Wainwright and McLean, 2017; Wiench et al., 2021). TB has been clinically used as a vital stain to highlight oral lesions. In addition, it has entered clinical trial as an antibacterial PS (Sridharan and Shankar, 2012; Singh et al., 2020). These examples of clinical application suggest TB’s excellent translational potential as an anticancer PS. However, TB also demonstrated a clear dark toxicity (Tremblay et al., 2002; Blazquez-Castro et al., 2009) and caused undesired side effects, which inevitably limited its administered dose. Recent studies have demonstrated that TB’s anticancer PDT effects could be improved by loading it into nanocarriers. The nanocarriers could help realize the cancer-targeted delivery through both enhanced permeation and retention (EPR) effects and the targeted nanomedicine (Xie et al., 2021). In addition, the controlled release of TB *via* nanocarriers effectively ameliorated its dark toxicity and improved the pharmacokinetics to favor cancer treatment (Lucky et al., 2015; Zhang and Tung, 2018). Until now, several nanomaterials, including gold, PLGA, and upconversion nanoparticles, have been exploited to deliver TB for anticancer PDT applications (Popp et al., 2012; Wang et al., 2020; Yang et al., 2021), while a delivery vehicle with intrinsic biological activities is of great significance to facilitate TB’s anticancer PDT.

DNA is a bioactive material featuring biocompatibility and flexibility to build different DNA materials for biosensing, imaging, and anticancer therapy (Chen et al., 2015; Lv et al., 2021). When combined with sequence design, nucleic acid therapeutics could be incorporated into DNA nanocarriers, thus potentially enhancing PDT efficiency (Li et al., 2019; Qu et al., 2022). For example, CpG DNA and G-quadruplex sequences, that act as immunoadjuvant and photosensitizer anchors, respectively, have been incorporated into DNA nanostructures through rolling circle amplification. This formed multifunctional DNA nanocarriers successfully realized the combination of PDT and immunotherapy for cancer (Wang et al., 2022). In fact, TB could stain the nucleus both in fixed and live cells due to its high affinity toward nucleic acids, especially DNA (Ilanchelian and Ramaraj, 2011; Singh et al., 2020). Inspired by TB’s noncovalent interactions with DNA, e.g., the direct intercalation and electrostatic interaction, in this study, we developed a self-assembled DNA nanogel to deliver TB for anticancer PDT. The TB-loaded DNA nanogel (DNA/TB NG) was comprehensively characterized, and the anticancer PDT effects were evaluated using the breast cancer cell line MCF-7. Compared with free TB, the prepared DNA/TB NG demonstrated excellent photostability, controlled TB-releasing behavior, and reduced dark toxicity. This DNA/TB NG potentially provided a facile and cost-effective strategy to deliver TB toward an improved anticancer PDT application.

## 2 Materials and methods

### 2.1 Materials

Herring sperm DNA (hsDNA, <50 bp, #D3159), cisplatin (#479306), o-phenylenediamine (OPDA, #P23938), and toluidine blue (TB, #T3260) were obtained from Sigma (St. Louis, MO, United States). The ROS probe 2’,7’-dichlorofluorescin diacetate (DCFDA) was purchased from Abcam (Cambridge, MA, United States). The MTS assay kit was purchased from Promega (Madison, WI, United States).

### 2.2 Synthesis of DNA/TB NG

Cisplatin (5 mg/mL) was dissolved in heated water to allow full dissolution. Then, 200 µL of cisplatin was mixed with 250 µL of TB (2 mg/mL in water) before adding 800 µL of hsDNA (5 mg/mL in water). The mixture was quickly heated at 90°C for 5 min and then cooled on ice for 5 min. Then, the DNA/TB NG was acquired by dialysis against pure water (MWCO = 3.5 kDa). The dialysis water was collected to quantify TB based on the absorbance peak at 633 nm using a standard curve. The TB content in DNA/TB NG was calculated by deducting the amount of TB in dialysis water from the input TB during the preparation. Cisplatin retained in the DNA/TB NG was quantified using a modified OPDA method as reported previously (Zhang et al., 2017). Briefly, the DNA/TB NG was diluted with 9% NaCl solution at a 10-fold rate. The diluted NG was mixed with an equal volume of 1.2 mg/mL OPDA in DMF, followed by 10 min heating at 100°C. Then, the absorbance at 705 nm of cisplatin–OPDA adducts was determined on a microplate reader (Tecan Group Ltd., Männedorf, Switzerland). The cisplatin content was correlated using a cisplatin standard curve with a concentration ranging from 0.625 to 20 μg/mL. The drug loading efficiency was defined as the weight of the encapsulated drug divided by the total NG weight. To prepare the blank DNA NG, hsDNA plus cisplatin only was heated and cooled following the same protocol used for the DNA/TB NG.

### 2.3 Characterization of DNA/TB NG

The diluted DNA/TB NG solution was dropped on a copper grid, left to dry, and then observed using a TEM (JEM-1400, JEOL, Tokyo, Japan). For negative staining, the DNA/TB NG on the copper grid was stained with 1.5% uranyl acetate for 2 min. To measure the size, at least 100 particles from TEM images (with negative staining) were counted. The DNA/TB NG was diluted with an appropriate volume of pure water, and then the dynamic light scattering (DLS) analysis was conducted on a Zetasizer Nano ZS90 (Brookhaven Instruments, Holtsville, NY, United States). The absorption spectra of DNA/TB NG were collected using a Cary 60 UV-Vis spectrophotometer (Agilent Technologies, Santa Clara, CA, United States). To measure the fluorescence spectra, aqueous solution of DNA/TB NG was excited at 620 nm, and emission ranging from 650 to 830 nm was recorded using a microplate reader (Tecan Group Ltd., Männedorf, Switzerland). In drug release analysis, 1 mL of free TB or DNA/TB NG solution was sealed in a dialysis tube and immersed in 80 mL of PBS. The dialysis was conducted in an incubator at 37°C with a rotation speed of 100 rpm. At pre-determined time points, PBS was collected out of the dialysis tube, and an equal volume of fresh PBS was replenished. The TB content in the collected PBS was quantified based on the absorbance, as described previously. Before and after the dialysis in PBS, the hydrodynamic size of DNA/TB NG was measured using a DLS sizer to monitor the stability.

### 2.4 Photostability of DNA/TB NG

To evaluate the photostability, free TB and DNA/TB NG solution in a transparent 96-well plate were kept in the dark or irradiated by an LED light source (660 nm, 25 mW/cm^2^) for 0, 2, 5, and 10 min. After each light irradiation, both the absorption and fluorescence spectra were monitored using the microplate reader.

### 2.5 Cell culture

The human breast cancer cell line MCF-7 was obtained from the Cell Resource Center of Chinese Academy of Medical Sciences and Peking Union Medical College (Beijing, China). The cells were cultured in RPMI 1640 medium supplemented with 10% FBS and 100 U/mL streptomycin and penicillin. Cells seeded in dishes or plates were kept at 37°C in a humidified incubator with 5% CO_2_.

### 2.6 Fluorescence microscopy and FACS analysis

Cells seeded in 8-well chamber µ-slides (ibidi, Martinsried, Germany) were treated with free TB and DNA/TB NG (8 μM TB) for 20 h. Then, the cells were incubated with Hoechst dye for 2 min to stain the nucleus. The cells were observed on an EVOS FL Auto microscope (Thermo Fisher Scientific, Waltham, United States) with a Cy5 channel for TB and a DAPI channel for Hoechst imaging. The intracellular TB fluorescence was quantified using Fiji/ImageJ software. For intracellular ROS analysis, cells were first treated with free TB and DNA/TB with the TB concentration set at 2 μM. Then, the cells were stained using the DCFDA probe following the manufacturer’s instructions. The cells were further irradiated by an LED light source (660 nm, 25 mW/cm^2^) for 10 min. Immediately after the light irradiation, DCFDA fluorescence from the cells was analyzed on a Gallios flow cytometer (Beckman Coulter, Brea, CA, United States).

### 2.7 Cell viability analysis

MCF-7 cells were seeded in a 96-well plate and incubated overnight to allow cell adhesion. To evaluate the viability of cells treated by blank DNA NG, cells were treated by the NG with a concentration ranging from 2.7 to 167 μg/mL for 48 h. To determine the dark and light toxicity of the DNA/TB NG, the cells were treated by free TB and DNA/TB NG with TB concentrations set at 0, 0.1, 0.4, 1, 2, and 4 µM. After 20 h of incubation, the cells in the PDT group were irradiated with an LED light source (660 nm, 25 mW/cm^2^) for 30 min. After 1 day, the cell viability was evaluated using the MTS assay kit following the manufacturer’s instructions. The viability of cells with neither TB nor light treatment was expressed as 100%.

### 2.8 Statistical analysis

All the results were expressed as the mean ± standard deviation (SD). Student’s t-test was used for statistical analysis, with a *p*-value <0.05 considered statistically significant.

## 3 Results and discussion

### 3.1 Preparation of DNA/TB NG

**FIGURE 1:**
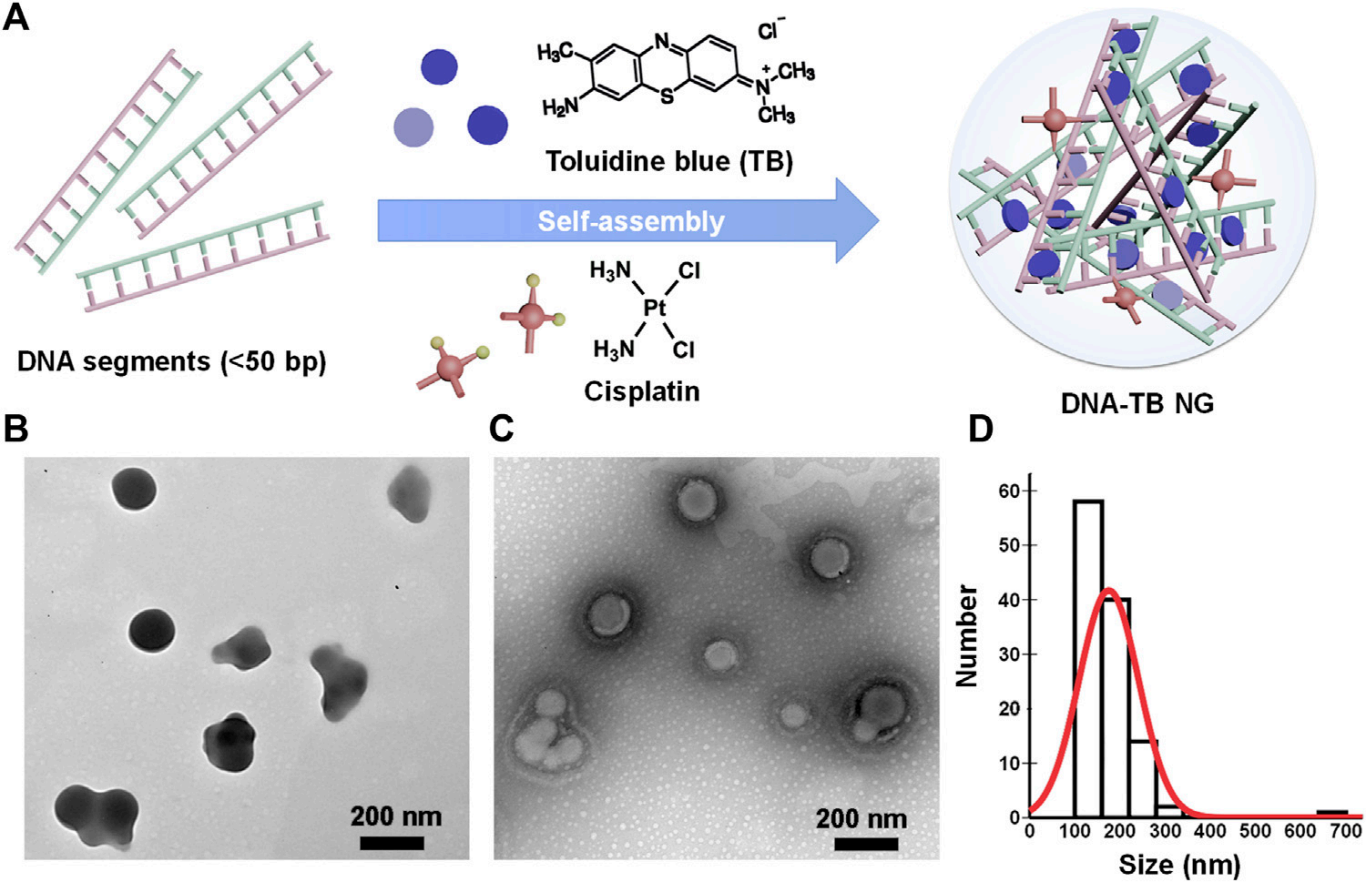
Preparation of DNA/TB NG. **(A)** Schematic illustration of the self-assembly of DNA/TB NG. **(B)** Representative TEM image (no staining). **(C)** Representative TEM image of the DNA/TB NG with negative staining. **(D)** Size distribution of DNA/TB NG calculated from TEM (with staining).

To prepare the self-assembled DNA nanogel, herring sperm DNA (hsDNA) segments (<50 bp) were selected as a model DNA since it is readily available and cost-effective and widely used for studying drug–DNA interactions (Sirajuddin et al., 2013; Xiong et al., 2021). Cisplatin, a common chemotherapeutic drug that could also act as a crosslinker to build different nanoparticles (Li et al., 2014; Zhang and Tung, 2017a; Wang et al., 2021; Sala et al., 2022), was utilized to construct the DNA nanogel, as previously demonstrated (Zhang and Tung, 2017b). A simple mixing of cisplatin, TB, and hsDNA, followed by a heating and cooling process, generated the DNA/TB NG (Figure 1A). As revealed by TEM with or without negative staining, the DNA/TB NG clearly presented as spherical nanostructures (Figures 1B, C). TEM size measurements suggested that the DNA/TB NG had a size of approximately 175.1 nm (Figure 1D). Dynamic light scattering (DLS) analysis revealed that the DNA/TB NG was negatively charged (−25.6 mV), and the hydrodynamic size was approximately 236.1 nm with a narrow distribution, as indicated by a polydispersity index (PDI) of approximately 0.155 (Table 1). This observed larger size from DLS measurements could have resulted from DNA/TB as a nanoscale hydrogel adsorbs water molecules and swells in aqueous solutions.

**TABLE 1:** DLS analysis and TB loading efficiency of DNA/TB NG.

	DNA/TB NG
Size	236.1 ± 24 nm
PDI	0.155 ± 0.05
Zeta potential	−25.6 ± 2.0 mV
TB loading efficiency	9.36% ± 0.63%
Cisplatin loading efficiency	0.14% ± 0.02%

### 3.2 Characterization of DNA/TB NG

**FIGURE 2:**
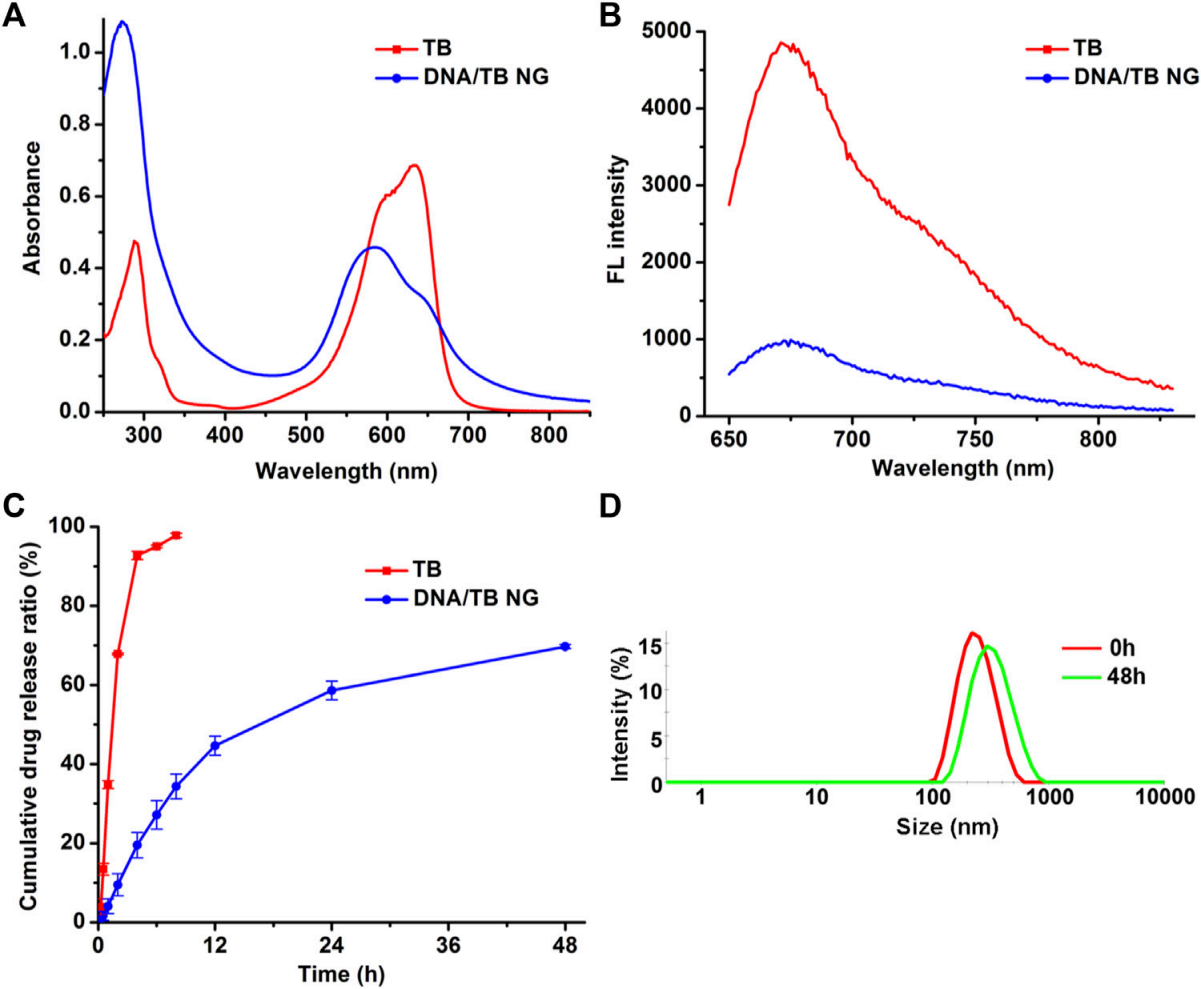
Spectra and drug release analysis of DNA/TB NG. **(A)** Absorption spectra and **(B)** fluorescence spectra of free TB and DNA/TB NG. The TB concentration was fixed at 20 μg/mL. **(C)** Drug release of TB in PBS at 37°C. **(D)** Size distribution of DNA/TB NG dispersed in PBS for 2 days (37°C).

In the UV-Vis-NIR absorption spectrum (Figure 2A), the appearance of a TB absorption peak in DNA/TB NG was found. The control nanogel without TB encapsulation (named blank DNA NG) only showed a peak at approximately 260 nm, which was inherent to DNA absorption (Supplementary Figure S1). The absorption of TB in DNA/TB NG demonstrated a blue shift (Figure 2A), which might have resulted from the binding between TB and DNA or part of TB aggregated within the NG. This phenomenon, together with that of TB fluorescence in NG, was quenched compared with free TB (Figure 2B), strongly suggesting that TB was successfully loaded in the NG. The TB loading efficiency in the NG was approximately 9.35%, while only 0.14% of cisplatin was found in DNA/TB NG (Table 1). Considering cisplatin’s role as chemotherapeutics, the biocompatibility of the blank DNA NG containing similar cisplatin content to DNA/TB NG was evaluated using the cell viability assay, and no observable cytotoxicity was found, with the NG content set up to 167 μg/mL (Supplementary Figure S1C). Given the trace amount of cisplatin retained in the NG, the contribution of cisplatin’s cytotoxicity for DNA/TB NG would be neglectable. As the controlled drug release played a pivotal role in determining nanomedicine’s therapeutic efficacy (Farokhzad and Langer, 2006; Zhao et al., 2012), the TB’s release curve in PBS buffer was evaluated at 37°C. The free TB could diffuse through the dialysis membrane and thus was released rapidly within 8 h (Figure 2C). Unlike free TB, the DNA/TB NG demonstrated a slowly releasing behavior within 48 h incubation. The DNA/TB NG appeared slightly swollen in PBS (Figure 2D), possibly due to the removal of TB, as approximately 70% of it had been released after 2 days. The absence of significant aggregation of DNA/TB NG in PBS buffer suggested that it would still behave as a nanoparticle even after being introduced into the biological environment.

### 3.3 Photostability of DNA/TB NG

**FIGURE 3:**
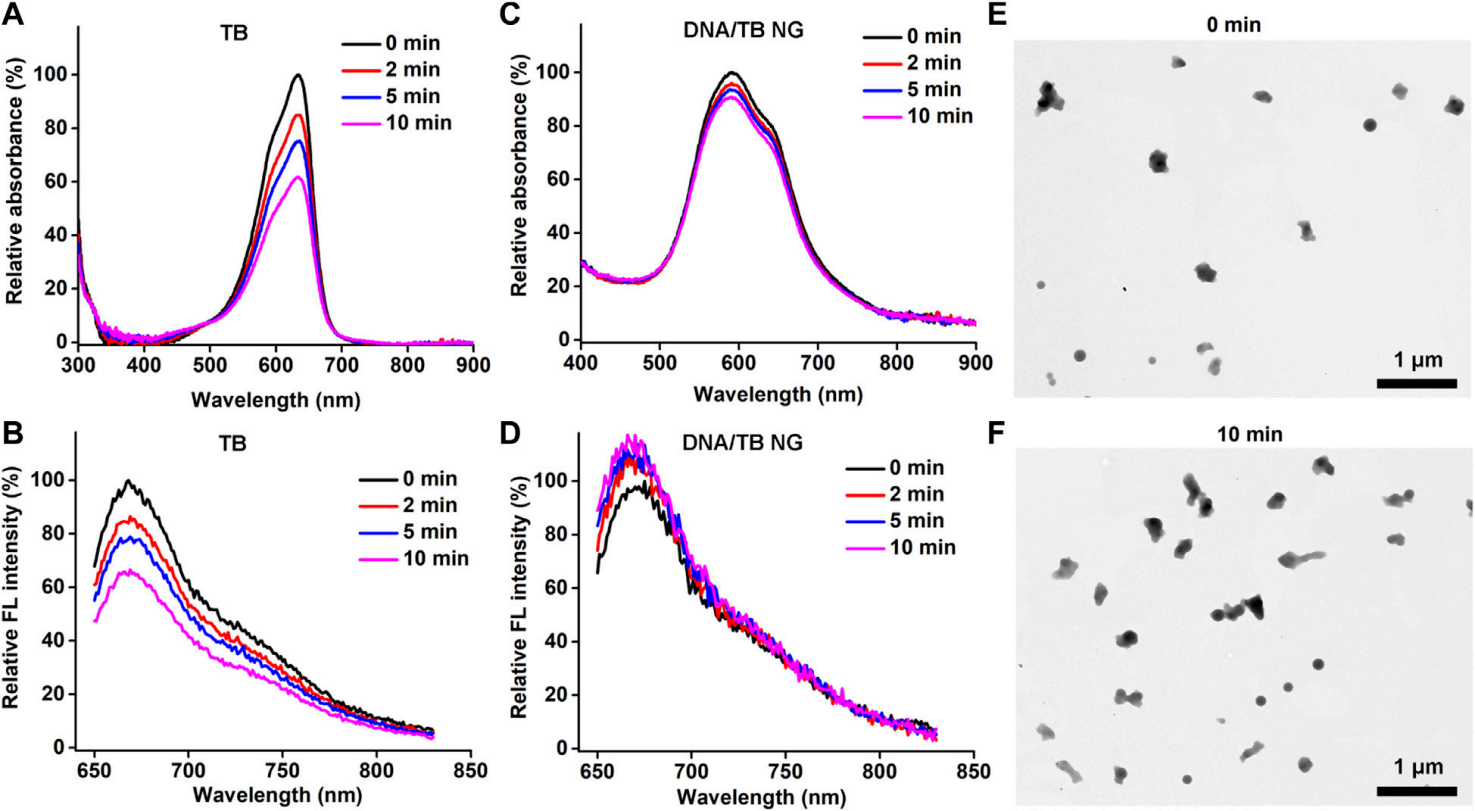
Photostability of DNA/TB NG. **(A)** Absorption and **(B)** fluorescence spectra of free TB after light irradiation at different minutes. **(C)** Absorption and **(D)** fluorescence spectra of DNA/TB NG. **(E, F)** Morphology of DNA/TB NG before **(E)** and after **(F)** 10 min light irradiation (660 nm, 25 mW/cm^2^).

Photostability is a key parameter for PSs. The light-stimulated ROS during the PDT process could also break the PS structure, thereby causing photobleaching (Yogo et al., 2005; Tasso et al., 2019). To evaluate the photostability of DNA/TB NG in comparison to free TB, the aqueous solution of both TB and DNA/TB was exposed to an LED light source (660 nm) for up to 10 min, and the absorption and fluorescence spectra were recorded. As shown in Figures 3A, B, the light exposure could clearly decrease TB absorption and fluorescence intensity in the case of free TB, while minimal photobleaching was found after the same treatment for DNA/TB NG (Figures 3C, D). The improved photostability of TB in DNA/TB NG was understandable, as different nanomaterials were proven to protect the encapsulated dye from photobleaching (Zhao et al., 2009; Zhang et al., 2021). The morphology of DNA/TB NG was also imaged using the TEM before and after the light irradiation. No structural disruption of NG was observed after the light stimulation (Figures 3E, F), thus supporting the favorable photostability of DNA/TB NG.

### 3.4 Cellular uptake *in vitro*

**FIGURE 4:**
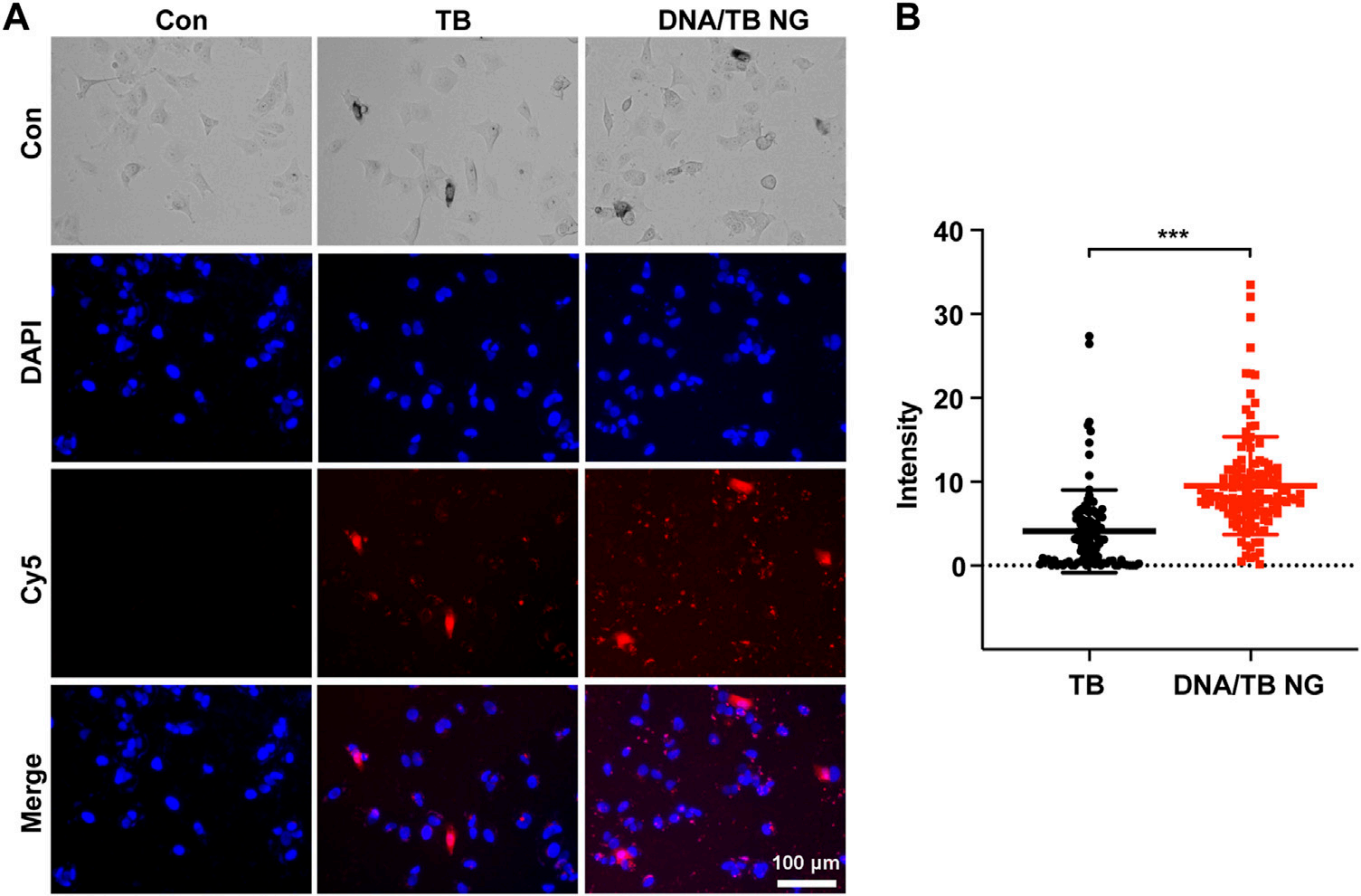
Fluorescent microscopy of MCF-7 cells treated with free TB and DNA/TB NG (8 µM of TB) for 20 h. **(A)** Representative fluorescence images. **(B)** Intracellular TB fluorescence intensity quantified from the fluorescence images.

Encouraged by the successful preparation of DNA/TB NG, the cellular uptake of DNA/TB and its intracellular localization were evaluated using the breast cancer cell line MCF-7. After 20 h of incubation with free TB, fluorescence microscopy found strong TB fluorescence in a minority of cells, while the fluorescence in the rest of the cells was very dim (Figure 4). In the case of DNA/TB-treated cells, heterogeneous fluorescence intensity in cells was also observed, while stronger fluorescence was presented in most of the cells than those treated with free TB (Figure 4B). This heterogeneous uptake of TB in cells might be dependent on the phenotypic and functional heterogeneity between cancer cells.

### 3.5 PDT effects *in vitro*

**FIGURE 5:**
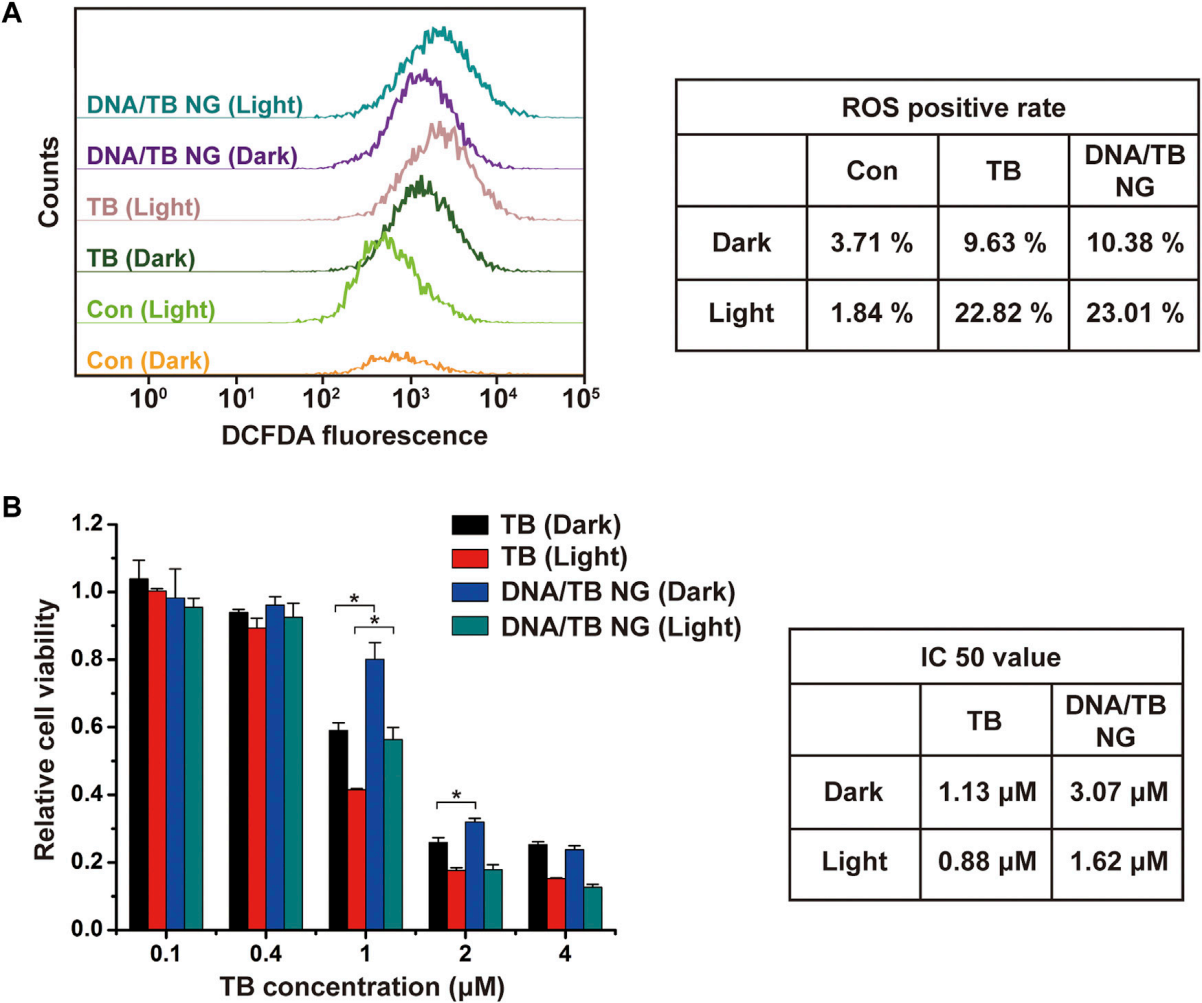
Comparison of PDT effects of TB and DNA/TB NG on MCF-7 cells. **(A)** FACS analysis of ROS in cells. The TB concentration was 2 µM. **(B)** Cytotoxicity of TB and DNA/TB NG with or without light irradiation.

In order to examine the PDT performance of DNA/TB NG, the ROS generation in cells was measured by DCFDA staining and flow cytometry (FACS). As demonstrated in Figure 5A, with the light irradiation, the ROS-positive rate of TB-treated cells rose from 9.63% to 22.82%, which was similar to that of DNA/TB NG with a ROS-positive rate that increased from 10.38% to 23.01%. The loading of TB in DNA/TB NG did not compromise the ROS generation in cells, as TB would be released following the degradation of DNA NGs. The dark and light toxicity were then evaluated with the presence and absence of light irradiation (660 nm), respectively. The dark toxicity of DNA/TB NG was weaker than that of free TB when the TB concentration was set at 1 and 2 µM (Figure 5B). With the presence of light, clear PDT toxicity was observed for both TB and DNA/TB NG in a concentration-dependent manner. Although DNA/TB NG elicited relatively less phototoxicity at 1 µM of TB, it could compete with free TB at higher TB concentrations. The light condition used here did not affect the cell viability of control cells (Supplementary Figure S2), suggesting that the observed phototoxicity was exclusively from the PS. The IC_50_ value of DNA/TB NG from dark to light was decreased by 47.2% (from 3.07 to 1.62 µM), while the decrease was 22.1% for free TB (from 1.13 to 0.88 µM). Since TB release in NG was relatively slower (Figure 2C), the DNA/TB NG generated less cytotoxicity in the dark, which is meaningful for advancing TB’s anticancer PDT application. More benefits of DNA NG were expected *in vivo* as EPR effects might help the accumulation of NGs at tumor sites (Zhang et al., 2014; Xie et al., 2021). Meanwhile, it should be noted that the hsDNA used here is a natural DNA extracted from Herring sperm, and thus potential contamination of endotoxin and immunogenic DNA sequence might exist (Kabbaj and Phillips, 2001; Urban et al., 2021), which would limit the current DNA/TB NG’s *in vivo* applications. Nevertheless, employing purified functional nucleic acids, such as immunoadjuvant CpG or siRNA, as templates can avoid the biocompatibility concerns of DNA NGs, which would also help fully exploit the biological activities of DNA NGs for TB delivery and anticancer PDT.

## 4 Conclusion

To summarize, in this study, we demonstrated that self-assembled DNA NG could be used as an effective TB delivery vehicle for anticancer PDT in the breast cancer cell line. The preparation of DNA/TB NG was assembled by cisplatin coordination and TB’s noncovalent binding with DNA, featuring with low cost, facile synthesis, and good biocompatibility. The acquired DNA/TB NG successfully enabled a controlled TB release, improved photostability, and reduced dark toxicity, thus providing a promising strategy to facilitate anticancer PDT application of TB. Considering the advantages of quick and facile preparation of DNA/TB NG, future efforts may focus on combining functional DNA such as targeting sequence and nucleic acid therapeutics to increase the selectivity and PDT efficacy of DNA/TB NGs toward cancers.

## Data Availability

The raw data supporting the conclusion of this article will be made available by the authors, without undue reservation.

## References

[B1] Agostinis P. Berg K. Cengel K. A. Foster T. H. Girotti A. W. Gollnick S. O. (2011). Photodynamic therapy of cancer: An update. CA Cancer J. Clin. 61 (4), 250–281. 10.3322/caac.20114 21617154 PMC3209659

[B2] Blazquez-Castro A. Stockert J. C. Sanz-Rodriguez F. Zamarron A. Juarranz A. (2009). Differential photodynamic response of cultured cells to methylene blue and toluidine blue: Role of dark redox processes. Photochem. Photobiol. Sci. 8 (3), 371–376. 10.1039/b818585a 19255678

[B3] Chen Y. J. Groves B. Muscat R. A. Seelig G. (2015). DNA nanotechnology from the test tube to the cell. Nat. Nanotechnol. 10 (9), 748–760. 10.1038/nnano.2015.195 26329111

[B4] Farokhzad O. C. Langer R. (2006). Nanomedicine: Developing smarter therapeutic and diagnostic modalities. Adv. Drug Deliv. Rev. 58 (14), 1456–1459. 10.1016/j.addr.2006.09.011 17070960

[B5] Ilanchelian M. Ramaraj R. (2011). Binding interactions of toluidine blue O with *Escherichia coli* DNA: Formation of bridged structure. J. Fluoresc. 21 (4), 1439–1453. 10.1007/s10895-010-0829-4 21344224

[B6] Kabbaj M. Phillips N. C. (2001). Anticancer activity of mycobacterial DNA: Effect of formulation as chitosan nanoparticles. J. Drug Target. 9(5), 317–328. 10.3109/10611860108998768 11770702

[B7] Li M. Tang Z. Lv S. Song W. Hong H. Jing X. (2014). Cisplatin crosslinked pH-sensitive nanoparticles for efficient delivery of doxorubicin. Biomaterials 35 (12), 3851–3864. 10.1016/j.biomaterials.2014.01.018 24495487

[B8] Li M. Y. Wang C. L. Di Z. H. Li H. Zhang J. F. Xue W. T. (2019). Engineering multifunctional DNA hybrid nanospheres through coordination-driven self-assembly. Angew. Chem. Int. Ed. 58 (5), 1350–1354. 10.1002/anie.201810735 30506904

[B9] Li X. S. Lovell J. F. Yoon J. Chen X. Y. (2020). Clinical development and potential of photothermal and photodynamic therapies for cancer. Nat. Clin. Pract. Oncol. 17 (11), 657–674. 10.1038/s41571-020-0410-2 32699309

[B10] Lucky S. S. Soo K. C. Zhang Y. (2015). Nanoparticles in photodynamic therapy. Chem. Rev. 115 (4), 1990–2042. 10.1021/cr5004198 25602130

[B11] Lv Z. Zhu Y. Li F. (2021). DNA functional nanomaterials for controlled delivery of nucleic acid-based drugs. Front. Bioeng. Biotechnol. 9, 720291. 10.3389/fbioe.2021.720291 34490226 PMC8418061

[B12] Marino-Ocampo N. Reyes J. S. Gunther G. Heyne B. Fuentealba D. (2022). Thiol-reacting toluidine blue derivatives: Synthesis, photophysical properties and covalent conjugation with human serum albumin. Dyes Pigm 201, 110225. 10.1016/j.dyepig.2022.110225

[B13] Pham T. C. Nguyen V. N. Choi Y. Lee S. Yoon J. (2021). Recent strategies to develop innovative photosensitizers for enhanced photodynamic therapy. Chem. Rev. 121 (21), 13454–13619. 10.1021/acs.chemrev.1c00381 34582186

[B14] Popp J. Al-Majmaie R. Drexler W. Alattar N. Zerulla D. Tuchin V. V. (2012). Toluidine blue O-conjugated gold nanoparticles for hotodynamic therapy of cultured colon cancer. Biophot. Photonic Solutions Better Health Care III 842722. 10.1117/12.921813

[B15] Qu Y. Shen F. Zhang Z. Wang Q. Huang H. Xu Y. (2022). Applications of functional DNA materials in immunomodulatory therapy. ACS Appl. Mat. Interfaces 14 (40), 45079–45095. 10.1021/acsami.2c13768 36171537

[B16] Sala L. Perecko T. Mestek O. Pinkas D. Homola T. Kocisek J. (2022). Cisplatin-cross-linked DNA origami nanostructures for drug delivery applications. ACS Appl. Nano Mat. 5 (9), 13267–13275. 10.1021/acsanm.2c02976

[B17] Singh S. Halder A. Sinha O. Chakrabarty N. Chatterjee T. Adhikari A. (2020). Spectroscopic studies on the biomolecular recognition of toluidine blue: Key information towards development of a non-contact, non-invasive device for oral cancer detection. Front. Oncol. 10, 529132. 10.3389/fonc.2020.529132 33194588 PMC7653096

[B18] Sirajuddin M. Ali S. Badshah A. (2013). Drug-DNA interactions and their study by UV-Visible, fluorescence spectroscopies and cyclic voltametry. J. Photochem. Photobiol. B 124, 1–19. 10.1016/j.jphotobiol.2013.03.013 23648795

[B19] Sridharan G. Shankar A. A. (2012). Toluidine blue: A review of its chemistry and clinical utility. J. Oral Maxillofac. Pathol. 16 (2), 251–255. 10.4103/0973-029X.99081 22923899 PMC3424943

[B20] Tasso T. T. Schlothauer J. C. Junqueira H. C. Matias T. A. Araki K. Liandra-Salvador E. (2019). Photobleaching efficiency parallels the enhancement of membrane damage for porphyrazine photosensitizers. J. Am. Chem. Soc. 141 (39), 15547–15556. 10.1021/jacs.9b05991 31490678

[B21] Tremblay J. F. Dussault S. Viau G. Gad F. Boushira M. Bissonnette R. (2002). Photodynamic therapy with toluidine blue in jurkat cells: Cytotoxicity, subcellular localization and apoptosis induction. Photochem. Photobiol. Sci. 1 (11), 852–856. 10.1039/b204385h 12659523

[B22] Urban L. A. Trinh A. Pearlman E. Siryaporn A. Downing T. L. (2021). The impact of age-related hypomethylated DNA on immune signaling upon cellular demise. Trends Immunol. 42 (6), 464–468. 10.1016/j.it.2021.04.005 33994111 PMC9650629

[B23] Wainwright M. McLean A. (2017). Rational design of phenothiazinium derivatives and photoantimicrobial drug discovery. Dyes Pigm 136, 590–600. 10.1016/j.dyepig.2016.09.015

[B24] Wang D. Liu J. Duan J. Ma Y. Gao H. Zhang Z. (2022). Photocontrolled spatiotemporal delivery of DNA immunomodulators for enhancing membrane-targeted tumor photodynamic immunotherapy. ACS Appl. Mat. Interfaces 14 (39), 44183–44198. 10.1021/acsami.2c12774 36165393

[B25] Wang H. Deng H. Gao M. Zhang W. (2021). Self-assembled nanogels based on ionic gelation of natural polysaccharides for drug delivery. Front. Bioeng. Biotechnol. 9, 703559. 10.3389/fbioe.2021.703559 34336811 PMC8322728

[B26] Wang S. Wei Z. Li L. Ning X. Liu Y. (2020). Luminescence imaging-guided triple-collaboratively enhanced photodynamic therapy by bioresponsive lanthanide-based nanomedicine. Nanomedicine 29, 102265. 10.1016/j.nano.2020.102265 32668297

[B27] Wang X. Q. Lei Q. Zhu J. Y. Wang W. J. Cheng Q. Gao F. (2016). Cucurbit[8]uril regulated activatable supramolecular photosensitizer for targeted cancer imaging and photodynamic therapy. ACS Appl. Mat. Interfaces 8 (35), 22892–22899. 10.1021/acsami.6b07507 27513690

[B28] Wiench R. Skaba D. Matys J. Grzech-Lesniak K. (2021). Efficacy of toluidine blue-mediated antimicrobial photodynamic therapy on Candida spp. A systematic review. Antibiot. (Basel) 10 (4), 349. 10.3390/antibiotics10040349 PMC8064486 33806003

[B29] Xie J. Wang Y. Choi W. Jangili P. Ge Y. Xu Y. (2021). Overcoming barriers in photodynamic therapy harnessing nano-formulation strategies. Chem. Soc. Rev. 50 (16), 9152–9201. 10.1039/d0cs01370f 34223847

[B30] Xiong Y. Li J. Huang G. Yan L. Ma J. (2021). Interacting mechanism of benzo(a)pyrene with free DNA *in vitro* . Int. J. Biol. Macromol. 167, 854–861. 10.1016/j.ijbiomac.2020.11.042 33181208

[B31] Yang Y. Tang T. Liu B. Tian J. Wu H. Liu Z. (2021). TB@PLGA nanoparticles for photodynamic/photothermal combined cancer therapy with single near-infrared irradiation. Int. J. Nanomed. 16, 4863–4871. 10.2147/IJN.S304713 PMC8291662 34295159

[B32] Yogo T. Urano Y. Ishitsuka Y. Maniwa F. Nagano T. (2005). Highly efficient and photostable photosensitizer based on BODIPY chromophore. J. Am. Chem. Soc. 127 (35), 12162–12163. 10.1021/ja0528533 16131160

[B33] Zhang Q. Jiang Q. Li N. Dai L. R. Liu Q. Song L. L. (2014). DNA origami as an *in vivo* drug delivery vehicle for cancer therapy. ACS Nano 8 (7), 6633–6643. 10.1021/nn502058j 24963790

[B34] Zhang W. Du B. Gao M. Tung C. H. (2021). A hybrid nanogel to preserve lysosome integrity for fluorescence imaging. ACS Nano 15 (10), 16442–16451. 10.1021/acsnano.1c05864 34612039

[B35] Zhang W. Tung C. H. (2017a). Cisplatin cross-linked multifunctional nanodrugplexes for combination therapy. ACS Appl. Mat. Interfaces 9 (10), 8547–8555. 10.1021/acsami.6b16500 28224786

[B36] Zhang W. Tung C. H. (2018). Real-time visualization of lysosome destruction using a photosensitive toluidine blue nanogel. Chem. - Eur. J. 24 (9), 2089–2093. 10.1002/chem.201705697 29314346 PMC6309271

[B37] Zhang W. Tung C. H. (2017b). Sequence-independent DNA nanogel as a potential drug carrier. Macromol. Rapid Commun. 38 (20), 1700366. 10.1002/marc.201700366 PMC6317892 28895266

[B38] Zhang W. Zhang Z. Tung C. H. (2017). Beyond chemotherapeutics: Cisplatin as a temporary buckle to fabricate drug-loaded nanogels. Chem. Commun. 53 (4), 779–782. 10.1039/c6cc08230k PMC5319414 27999837

[B39] Zhao B. Yin J. J. Bilski P. J. Chignell C. F. Roberts J. E. He Y. Y. (2009). Enhanced photodynamic efficacy towards melanoma cells by encapsulation of Pc4 in silica nanoparticles. Toxicol. Appl. Pharmacol. 241 (2), 163–172. 10.1016/j.taap.2009.08.010 19695274 PMC2783992

[B40] Zhao Y. X. Shaw A. Zeng X. Benson E. Nystrom A. M. Hogberg B. (2012). DNA origami delivery system for cancer therapy with tunable release properties. ACS Nano 6 (10), 8684–8691. 10.1021/nn3022662 22950811

