# Supplementary material for: Facile preparation of toluidine blue-loaded DNA nanogels for anticancer photodynamic therapy
Source: Front Bioeng Biotechnol. 2023 Apr 18;11:1180448. doi: 10.3389/fbioe.2023.1180448 (PMC10151483; doi:10.3389/fbioe.2023.1180448)
Supplement: Supplementary file 1 [file DataSheet1.docx]

Supplementary Material


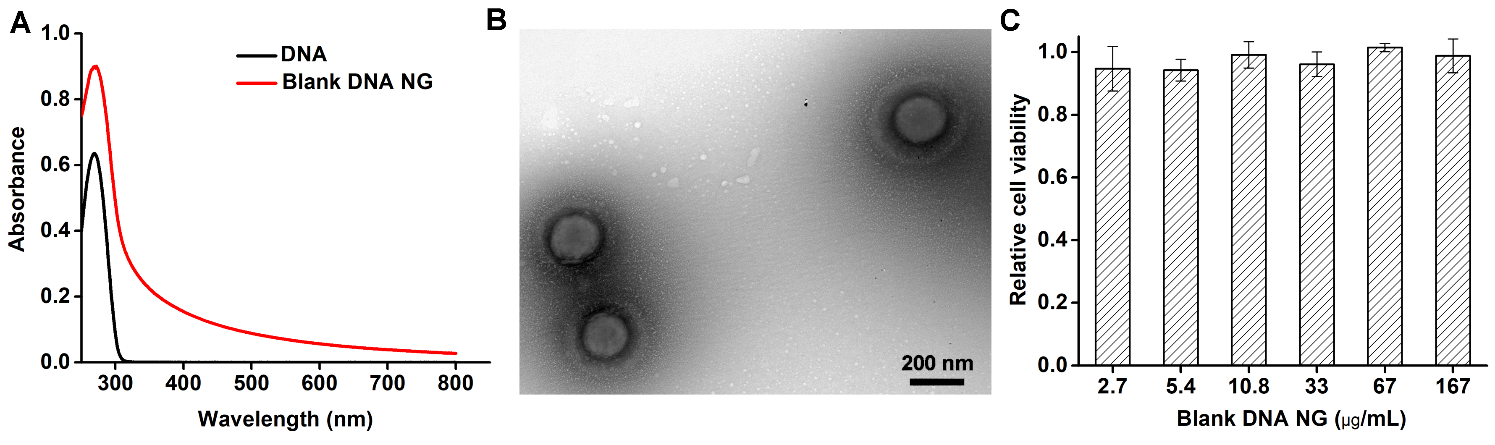


**Figure S1.** Characterization of the blank DNA NG. (A) The UV-Vis-NIR absorption spectra of blank DNA NG and DNA only. (B) TEM observation of blank DNA NG with negative staining. (C) Cell viability of MCF-7 cells treated by blank DNA NG for 48 hours.


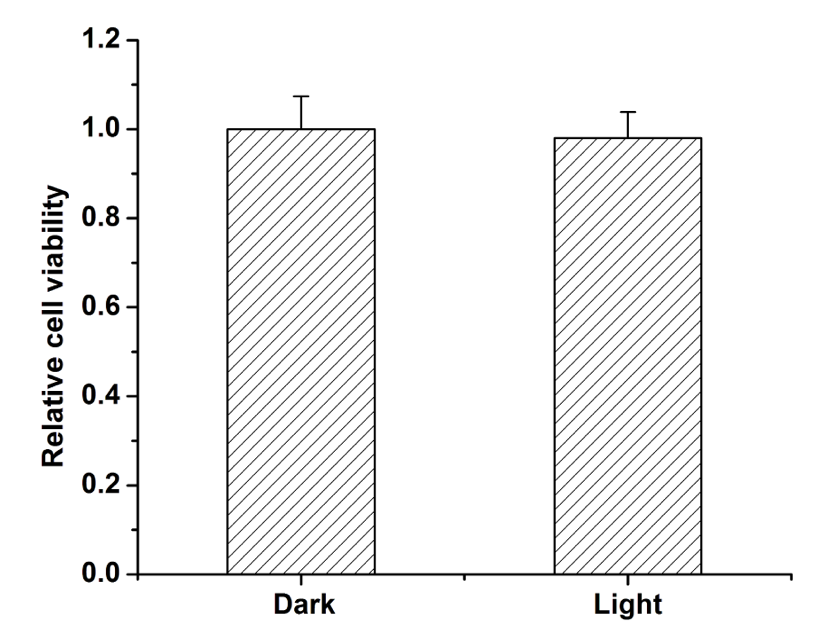


**Figure S2.** Effect of light on control cells determined by cell viability assay. Light irradiation: 660 nm, 25 mW/cm^2^, 30 minutes.
